# Fish Peroxiredoxins and Their Role in Immunity

**DOI:** 10.3390/biology4040860

**Published:** 2015-11-27

**Authors:** Yulema Valero, Francisco J. Martínez-Morcillo, M. Ángeles Esteban, Elena Chaves-Pozo, Alberto Cuesta

**Affiliations:** 1 Centro Oceanográfico de Murcia, Instituto Español de Oceanografía (IEO), Carretera de la Azohía s/n, Puerto de Mazarrón, Murcia 30860, Spain; E-Mails: yulema.valero@mu.ieo.es (Y.V.); elena.chaves@mu.ieo.es (E.C.-P.); 2 Fish Innate Immune System Group, Department of Cell Biology and Histology, Faculty of Biology, Campus Regional de Excelencia Internacional “Campus Mare Nostrum”, University of Murcia, Murcia 30100, Spain; E-Mails: javimartinez12@hotmail.com (F.J.M.-M.); aesteban@um.es (M.Á.E.)

**Keywords:** peroxiredoxins, fish, immunity, antioxidant enzymes, natural killer enhancement factor, virus

## Abstract

Peroxiredoxins (Prxs) are a family of antioxidant enzymes that protect cells from oxidative damage. In addition, Prxs may act as modulators of inflammation, protect against cell death and tumour progression, and facilitate tissue repair after damage. The most studied roles of Prx1 and Prx2 are immunological. Here we present a review on the effects of some immunostimulant treatments and bacterial, viral, or parasitic infections on the expression of fish Prxs at the gene and/or protein level, and point to their important role in immunity. The Prxs show antioxidant activity as well as a protective effect against infection. Some preliminary data are presented about the role of fish Prx1 and Prx2 in virus resistance although further studies are needed before the role of fish Prx in immunity can be definitively defined.

## 1. Introduction

**Figure 1:**
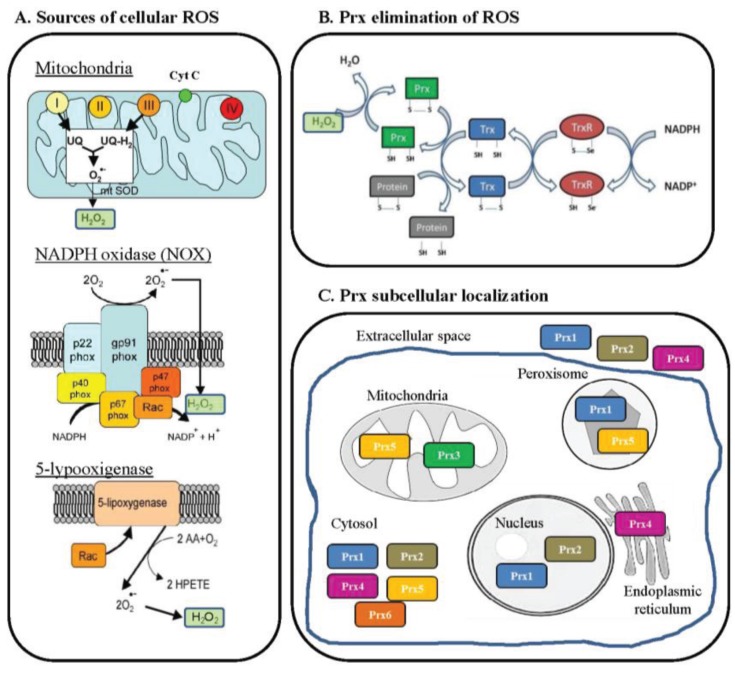
Aspects related with peroxiredoxins and the metabolism of reactive oxygen species (ROS). (**A**) Major sources of H_2_O_2_ are mitochondria, the NADPH oxidase (NOX) complex of phagocytic cells, and 5-lipoxygenase (reviewed by [2]). In the mitochondria, electrons from the electron transport chain are used to form O_2_^−^ at the level of complex I and III, before being converted by mitochondrial superoxide dismutase (SOD) into H_2_O_2_, which can cross mitochondrial membranes to reach the cytoplasm. Regarding the NOX complexes, they are present in both professional phagocytic cells (macrophages, neutrophils, and eosinophils) and non-phagocytic cells and play a crucial role in different diseases. The NOX of non-phagocytic cells is constitutively active, producing a very low level of ROS and increasing both its activity and ROS generation in response to a number of factors and conditions. Finally, 5-lipoxygenase (5-LOX) is a mixed function oxidase involved in the synthesis of leukotrienes from arachidonic acid in response to essentially the same stimuli that are able to stimulate NOX, particularly growth factors and cytokines. The last mediators lead to membrane ruffling and the generation of superoxide, and then H_2_O_2_, through the intervention of the small GTPase Rac1 and a SOD isoform. (**B**) Mechanism of action of the thioredoxin (Trx) redox system (reviewed by [3]). Reduced Trx catalyses the reduction of disulfides (S-S) within oxidized cellular proteins, such as peroxiredoxin (Prx). In this process Trx becomes oxidized, is then reduced by thioredoxin reductase (TrxR) at the expense of NADPH. (**C**) Regarding their localization, Prx proteins can be nuclear (Prx1 and Prx2), mitochondrial (Prx3 and Prx5), peroxisomic (Prx1 and Prx5), located in the endoplasmic reticulum (Prx4), cytosolic (Prx1, Prx2, Prx4–6), or extracellular (Prx1, Prx2 and Prx4). Cyt C, cytochrome C; UQ, coenzyme Q or ubiquinone; mtSOD, mitochondrial superoxide dismutase; NADP^+^, nicotinamide adenine dinucleotide phosphate; NADPH, reduced NADP^+^; phox, phagocyte oxidase; Rac, Ras-related C3 botulinum toxin substrate; AA, arachidonic acid; HPETE, hydroperoxyeicosatetraenoic acid; Prx, Peroxiredoxin; Trx, Thioredoxin; TrxR, Thioxiredoxin reductase. Modified from [2,3,4].

One of the most topical subjects in Cell Biology and Physiology is the balance between the production and elimination of reactive oxygen species (ROS)—it is known as oxidative stress when this balance shifts to ROS production—and their role in aspects such as aging, cancer, stem cell, toxicology or immunity. ROS are mainly produced in eukaryotic living cells by mitochondria, as a result of an aerobic respiration process, and their production depends on the cell and tissue types and physiological situations (Figure 1) [1,2,3,4]. Oxygen is rapidly reduced by electrons formed during mitochondrial respiration and converted into several very reactive and unstable ions, such as superoxide anion (O_2_^−^), peroxide (O_2_^−2^), hydroxyl radical (HO^−^), and singlet oxygen (O_2_*). ROS are also produced by the NADPH oxidase (NOX) complexes present in cell membranes, mitochondria, peroxisomes, and endoplasmic reticulum (Figure 1), with greatest importance in macrophages and neutrophils after activation by immune stimuli. In addition, there are many other enzymes that produce cellular ROS, including 5-lypoxigenase, xanthine oxidase, nitric oxide synthase, cyclooxygenase, other NAD(P)H dependent oxido-reductases, glycolate oxidases, d-amino oxidases, ureate oxidases, fatty acid-CoA oxidases, l-α-hydroxyacid oxidases, or lysyl oxidase [2]. To eliminate the excess of ROS produced during many physiological and pathological processes, which can be harmful for cells, the mitochondria, and cells in general, possess numerous ROS defence or antioxidant systems. It should be mentioned here that the true source of oxidative stress is not ROS generation *per se*, but a spatiotemporal imbalance of ROS production and detoxification [1]. There are two main antioxidant enzymes involved in ROS scavenging: (i) superoxide dismutase (SOD), which rapidly converts the very unstable superoxide into the more stable hydrogen peroxide (H_2_O_2_); and (ii) catalase (CAT), which catalyses the reaction 2 H_2_O_2_ → H_2_O + O_2_ [1]. However, apart from the CAT enzyme, other antioxidant systems are present in cells and are equally important for the control of the cellular redox potential. Among them, thioredoxins (Trxs), glutaredoxins (Grxs), and peroxiredoxins (Prxs) have been characterized as electron donors, guardians of the intracellular redox state, and “antioxidants” (Figure 1) [1,2]. A number of excellent reviews have summarized much of the work carried out on the structure, function, and biology of Prxs in the ROS balance [5,6,7,8].

In addition, Prxs are able to directly interact with some proteins, thereby affecting other cellular functions, such as apoptosis, proliferation, iron metabolism, and the development and functioning of many animal tissues, organs, and systems [2]. Among these functions, it was observed that tumour and virus-infected cells were able to secrete Prxs, which bind to Toll-like receptor 4 (TLR4), linking peroxiredoxin proteins with the immune response [9,10]. Since then, it has been documented that Prxs may act as modulators of inflammation in pathogen infection and in protection against cell death, tissue repair after damage, and tumour progression [9,10]; the present review will focus on the role of peroxiredoxins in the immune response.

Fish are the largest group of vertebrates, with more than 31,000 species, and the first group in vertebrate evolution, making them very attractive models for animal evolution studies due to their key position. In addition, some fish species are used as models in biomedicine and many more are produced by the aquaculture industry for human consumption. The aim of this review is to summarize present day knowledge of Prxs in fish and their relation to fish immunity.

## 2. Peroxiredoxins in Mammals

### 2.1. General Description

Peroxiredoxin (Prx or Prdx), first described in yeast [11], is a family of antioxidant enzymes that protect cells from oxidative damage by reducing H_2_O_2_, peroxynitrite, and lipid peroxidation, as well as by scavenging thiyl radicals [7,11]. They catalyse the reduction of peroxides (Figure 1) and alkyl peroxides with the help of thioredoxin, therefore receiving the name thioredoxin peroxidases. Prx proteins, with 20–30 kDa, are present in all living organisms with multiple genes and protein isoforms. In vertebrates, the family is formed of six members, Prx1 through Prx6, and may represent up to 1% of total soluble cellular proteins. After catalase, they are probably the most important hydrogen peroxide-scavenging enzymes in cells. In fact, Prx1–4 or Prx6 knockout mice showed increased ROS levels, but were viable, demonstrating the importance of Prxs in ROS scavenging [2], but also that they are not exclusively involved in the maintenance of the cellular redox balance.

Depending on their structure and catalytic domains, Prxs can be divided into three subgroups: true 2-cysteines (Cys) (Prx1–Prx4), atypical 2-Cys (Prx6), and 1-Cys (Prx5) Prx proteins [7,12]. They also show differential compartmentalization (Figure 1): Prx1 is mainly localized in the cytosol, the nucleus, and peroxisomes, but is also secreted and found in serum; Prx2 is present in the cytosol and the nucleus, and, due to its extracellular position, also binds to cell membranes; Prx3 is located exclusively in mitochondria; Prx4 is found in the cytosol and the endoplasmic reticulum, and also outside the cell; Prx5 is localized in the cytosol, mitochondria, and peroxisomes; Prx6 is located in the cytosol, vesicles, and lysosomes (Figure 1) [2].

### 2.2. Roles in Immunity

Besides their functions in oxidative metabolism, Prxs may act as modulators of inflammation in pathogen infection and in protection against cell death, tissue repair after damage, and tumour progression [9,10]. With respect to immunity, the most studied are Prx1 and Prx2 (also known as natural killer enhancing factor (NKEF)-A and NKEF-B, respectively). First reports identified NKEFs as cytosolic proteins of the human red blood cells that augment the natural killer (NK) cell cytotoxic activity against the K562 tumour cell line [13]. Subsequent reports on the production of recombinant human Prx1 and Prx2 demonstrated that only the Prx1, in its reduced form, was able to enhance the NK activity, but it failed to act on the activity of lymphokine-activated killer (LAK) cells [13,14]. Moreover, the expression of both Prx1 and Prx2 by human CD8+ T-cells was increased at both the gene and protein levels by human immunodeficiency virus (HIV-1) infection, while cells transfected with either of the genes were refractory to HIV-1 viral infection [15]. Furthermore, Prx1 expression is augmented in various cancers [16] and some studies have also demonstrated that Prx1 can be secreted by some cancer cells, possibly *via* a non-classical secretory pathway [17].

Prx function is also linked to inflammatory processes [9,10]. The redox potential regulates some redox-sensitive transcriptional factors, such as nuclear factor kappaB (NF-κB) and nuclear factor E2-related factor 2 (Nrf2). Among Nrf2 targets, Prx1 and Prx6 are especially important in the initiation of acute inflammation. Prx 1–6 are intrinsic ligands for Toll-like receptor 4 (TLR4) [18], which in turn activates NF-κB, leading to the induction of pro-inflammatory cytokines [19] and cyclophilins [20,21], while Prx1 also binds to macrophage migration inhibitory factor (MIF) [22]. The Prxs released from necrotic and injured cells in the brain after cerebral infarction aggravated tissue damage through interaction with TLR4 [18,19]. In addition, Prx1 and Prx2 are released upon stimulation with tumour growth factor-β (TGFβ), interleukin-1β (IL-1β), lipopolysaccharide (LPS) and/or tumour necrosis factor-α (TNF-α) [9,10]. Moreover, Prx6 has both peroxidase and phospholipase A2 activities and plays an important role in the cytokine-induced activation of NADPH oxidase-2, which enhances activation of NF-κB and cytosolic Ca^2+^-dependent phospholipase A2, leading to the production of prostaglandins [23,24,25]. Moreover, nuclear Prx1 and Prx2 interact with transcription factors such as NF-κB [26], c-Myc [27] and androgen receptor (AR) [28], affecting their ability to regulate gene expression, while in the cytoplasm those Prxs have anti-apoptotic functions through direct or indirect interactions with the key apoptosis regulators, ASK1 [29], p66Shc [30] and GSTpi/JNK [31].

## 3. Peroxiredoxins in Fish

### 3.1. Description and Presence of Fish Prxs

In teleost fish, orthologues of all the *prx* genes have been found and the gene sequences of one or several *prxs* have been identified and studied in rainbow trout (*Oncorhynchus mykiss*) [32], ayu (*Plecoglossus altivelis*) [33,34], miiuy croaker (*Miichthys miiuy*) [35,36], large yellow croaker (*Pseudosciaena crocea*) [37], lamprey (*Lampetra japonica*) [38], turbot (*Scophthalmus maximus*) [39], spotted green pufferfish (*Tetraodon nigroviridis*) [40], Japanese flounder (*Paralichthys olivaceus*) [41], bluefin tuna (*Thunnus maccoyii*) [42], yellowtail kingfish (*Seriola lalandi*) [43], common carp (*Cyprinus carpio*) [44], channel catfish (*Ictalurus punctatus*) [45], gilthead seabream (*Sparus aurata*) [46], European sea bass (*Dicentrarchus labrax*) [47], Atlantic salmon (*Salmo salar*) [48], or rock bream (*Oplegnathus fasciatus*) [49], as well as in genomic studies with other fish species. One of the most complete characterizations of fish *prxs* genes was carried out in gilthead seabream [46], in which the six *prxs* were, for the first time in fish, identified together; furthermore, their distribution and regulation by parasite infection and dietary fish-oil substitution were analysed. Based on these results, the greatest divergence among vertebrate Prx1–4 resides in the N-terminus, although there is strict conservation of amino acid residues surrounding the N- and C-terminal catalytic Cys residues. As in mammals, the C-terminal region of the gilthead seabream Prx5 is smaller than those of true 2-Cys Prx enzymes and conserves the alternative Cys residue at the C-terminus, belonging thus to the atypical 2-Cys Prx subclass. On the other hand, in a wide range of fish species, Prx6 only contains the N-terminal Cys residue, a Prx isoform unequivocally recognized as a member of the 1-Cys Prx subclass. These gene characteristics are conserved and similarly present in all studied fish species.

The genomic organization of fish *prxs* has been little studied. However, it has been seen that the *prx1* genome sequence contains five exons and five introns in miiuy croaker [50], while *prx2* contains five exons and four introns in miiuy croaker and Atlantic cod (*Gadus morhua*), six exons and five introns in Japanese medaka (*Oryzias latipes*), and five exons and five introns in pufferfish [36,40].

However, independent of the genomic structure, the mRNA transcription showed very similar properties in all fish species. Transcription of *prxs* is ubiquitously distributed in all tissues but differs among fish tissues, isoforms and fish species. For example, Prxs are mainly expressed in the European sea bass brain and blood [47], in seabream liver [46], in rainbow trout liver and red blood cells [51], in pufferfish liver and gut [40], and in the peripheral blood and gills of common carp [44]. Of the Prx forms, *prx3* was the most expressed gene in miiuy croaker [35] and *prx4* in the gilthead seabream [46], but further studies are necessary in this respect. It is unclear whether this differential expression has any functional consequence or affects the balance of important factors involved in the production of different Prxs, since they are probably relevant for tissue formation [14].

Compared with genetic studies, characterization at the protein level has received much less attention. Prx proteins have also been identified and/or isolated in proteomic studies in several fish species, where their role has been related to oxidative stress and immunity [37,52,53,54,55]. In addition, a few studies have used antibodies to evaluate fish Prx protein localization and abundance. In rainbow trout, Prx1 (although the antibody cross-reaction with other Prxs, mainly Prx2, cannot be discounted) production was shown to increase upon *in vitro* or *in vivo* infection by using immunohistochemistry, western blot, and ELISA techniques [56,57]. Moreover, immunohistochemical studies found that cells resembling T-cells and macrophages were Prx1-positive in rainbow trout [56] while red blood and some epithelial cells were Prx2-positive in common carp [44]. Moreover, in both cases the number of cells and staining intensity increased upon infection, indicating increases at protein level.

Finally, the Prx function in fish has been mainly studied after exposure to immune *stimuli* (see Table 1) and very little information is related to its primordial antioxidant role. Thus, fish Prx expression, at either gene or protein level, is regulated by LPS treatment, pathogens including bacteria, virus and parasites, oxidative stress, diet or management stress [33,34,35,36,38,39,40,44,46,47,48,49,52,54,55,56,57,58,59]. Interestingly, most studies have confirmed an increase in Prx1/Prx2 expression after viral infection [44,52,57,58,59] but very few have correlated increases in Prx1/Prx2 (NKEFs) gene expression with augmented innate cytotoxic activity [57,59,60,61].

Most of the information available for teleost fish is derived from gene transcription levels or protein production following infection, and has mainly concerned Prx1 and Prx2. Unfortunately, very few data are available about the direct effect of Prx on the fish immune response (see Table 2), which could be the subject of future research. Moreover, in some papers, they have simply been referred to as NKEFs and no attempt has been made to ascribe them to either Prx1 or Prx2. In such cases, we have tried to re-name accordingly based on the gene sequences now available in databases.

### 3.2. Prx1 (NKEF-A) in Fish Immunity

In mammals, Prx1 is the most studied peroxiredoxin and it is clearly connected with inflammation, tissue repair after damage and tumour progression. In fish, the characterization of Prx1 has not been so exhaustive but covers most of the information related to fish immunity (Table 1 and Table 2). Unfortunately, there has been no mechanistic evaluation of fish Prx1 and almost all the information comes from the *prx1* gene expression recorded after fish immune stimulation or infection. Thus, the incubation of head-kidney leucocytes (HKLs) *in vitro* with mitogens (concanavalin A (ConA), phytohemagglutinin (PHA)) failed to alter the transcription of *prx1* in gilthead seabream or European sea bass [47].

**Table 1:** Summary of the studies showing the regulation of peroxiredoxin genes (*prx*) or protein (Prx) expression after common immune stimuli in fish.

Fish Species	Stimulant		Effect	References
Gilthead seabream (*Sparus aurata*)	Mitogens	ConA, PHA	≈ *prx1*, *prx2* in HKLs	[47]
	PAMPs	LPS	≈ *prx1*, *prx2* in HKLs	
		CpG ODNs	↑ *prx1*, *prx2* in HKLs	
		Poly I:C	↑ *prx2* in HKLs	
	Bacteria	*V. anguillarum*	↑ *prx1* in HKLs	
		*P. damselae*	≈ *prx1*, *prx2* in HKLs	
	Virus	NNV	≈ *prx1*, *prx2* in HKLs	
			↑ *prx1*, *prx2* *in vivo*	
	Parasite	*E. leei*	↑ *prx1*, *prx2*, *prx3*, *prx5* in exposed fish and parasite-free	[46]
			↓ *prx1*, *prx2*, *prx3*, *prx, prx6* in parasitized fish	
			≈ *prx4*	
European sea bass (*Dicentrarchus labrax*)	Mitogens	ConA, PHA	≈ *prx1*, *prx2* in HKLs	[47]
	PAMPs	LPS	≈ *prx1*, *prx2* in HKLs	
		CpG ODNs	↑ *prx1*, *prx2* in HKLs	
		Poly I:C	↑ *prx1*, *prx2* in HKLs	
	Bacteria	*V. anguillarum*	≈ *prx1*, *prx2* in HKLs	
		*P. damselae*	≈ *prx1*, *prx2* in HKLs	
	Virus	NNV	≈ *prx1*, *prx2* in HKLs	
			↑ *prx2* *in vivo*	
Japanese flounder (*Paralichthys olivaceus*)	Bacteria	*A. hydrophyla*	↑ *prx1* in spleen	[62]
		*E. tarda*	↑ *prx1* and Prx1 in spleen	[63]
Channel catfish (*Ictalurus punctatus*)	PAMPs	LPS	↑ *prx1* in spleen	[45]
Spotted green pufferfish (*Tetraodon nigroviridis*)	PAMPs	LPS	↑ *prx1, prx2* in spleen	[40]
Common carp (*Cyprinus carpio*)	Virus	SVCV	↑ *prx1* and *prx2* in PBLs	[44]
			↓ *prx1* in gills	
			↓ *prx2* in HK	
			↑ Prx2	
Lamprey (*Lampetra japonica*)	PAMPs	LPS	↑ *prx2* in spleen	[38]
Rainbow trout (*Oncorhynchus mykiss*)	PAMPs	LPS	≈ Prx1 in MØ	[56]
			↑ Prx1 in RTS11	
		Zymosan	≈ Prx1 in MØ	
	Bacteria	*V. ordalii*	↑ Prx1 in MØ	
	Virus	VHSV	↑ *prx1* in B, HK	[58]
			↑ *prx1* in RTS11	[57]
			↑ *prx1* in PBLs	[59]
		ISAV	≈ Prx1 in RTS11	[56]
	Parasite	*C. rogercresseyi*	↑ Prx1 in spleen, HK	
	Vaccine	VHSV DNA vaccine + infection	↑ *prx1*	[58]
Atlantic salmon (*Salmo salar*)	Virus	IHNV	↓ Prx2	[52]
	Parasite	*N. perurans*	↓ *prx1* in gill	[48]
Large yellow croaker (*Pseudosciaena crocea*)	Vaccine	*V. alginolyticus*, *V. parahemolyticus*, and *A. hydrophila*	↑ Prx1, Prx2,Prx4 in spleen	[37]
Rock bream (*Oplegnathus fasciatus*)	PAMPs	Poly I:C	↑ *prx6* in liver	[49]
	Virus	Iridovirus	↑ *prx6* in liver	
Miiuy croaker (*Miichthys miiuy*)	Bacteria	*V. anguillarum*	↑ *prx2*, *prx3*, *prx4*, *prx5* in kidney, spleen	[35,36]
Ayu (*Plecoglossus altivelis*)	Bacteria	*V. alginolytics*	↑ *prx2* in gut	[33]
		*A. hydrophila*	↑ *prx2* in all tissues	
			↑ Prx2 in liver	
Zebrafish (*Danio rerio*)	Bacteria	*A. hydrophila*	↑ *prx3* and *prx5* in gill	[55]
			↑ Prx3 and Prx5 in gill	
Turbot (*Scophthalmus maximus*)	PAMPs	Poly I:C	↑ *prx6* in liver, spleen	[34]
	Bacteria	*V. anguillarum*	↑ *prx6* in liver, spleen	
			↑ *prx2*	[39]
		*S. iniae*	↑ *prx6* in liver, spleen	[34]

Abbreviations used: PAMPs, pathogen-associated molecular patterns; ConA, concanavalin A; PHA, phytohemagglutinin; CpG ODN, unmethylated CpG oligodeoxynucleotides; LPS, lipopolysaccharide; PBL, peripheral blood leucocytes; HK, Head-kidney; HKL, head-kidney leucocytes; MØ, macrophages; ISAV, infectious salmon anemia virus; SVCV, spring viremia of carp virus; VHSV, viral haemorrhagic septicaemia virus; NNV, nodavirus; IHNV, infectious hematopoietic necrosis virus.

**Table 2:** Summary of the functional studies evaluating the role of fish peroxiredoxins.

Fish Species	Prx	Approximation	Effects Observed	References
European sea bass (*Dicentrarchus labrax*)	Prx1	Expression plasmids	↓ NNV expression in brain	This study
	Prx2			
Atlantic salmon (*Salmo salar*)	Prx1	Recombinant	Antioxidant defence	[48]
Japanese flounder (*Paralichthys olivaceus*)	Prx1	Expression plasmids	↓ bacterial infection	[63]
		knockdown	↓ bacterial resistance	
Lamprey (*Lampetra japonica*)	Prx2	Recombinant	Antioxidant defence	[38]
			Protected DNA from *in vitro* oxidation	
Large yellow croaker (*Pseudosciaena crocea*)	Prx4	Recombinant	Antioxidant defence	[37]
		Prx4 injection	↓ NF-κB activity	
			↓ *tnfa*, *chemokines*	
			↑ *il10*	
			↑ bacterial resistance	
		Knockdown by siRNA	↑ NF-κB activity	
			↑ *tnfa*, *chemokines*	
			↓ *il10*	
			↓ bacterial resistance	
		Prx4 without the N-terminal motif	↓ NF-κB activity	[64]
			↓ bacterial resistance	
			No antioxidant function	
Rock bream (*Oplegnathus fasciatus*)	Prx6	Recombinant	Protected DNA from *in vitro* oxidation	[49]
Turbot (*Scophthalmus maximus*)	Prx6	Recombinant	Protected hepatocytes from peroxide treatment	[34]

The regulation by several bacterial pathogen-associated molecular patterns (PAMPs) such as unmethylated CpG oligodeoxynucleotides (CpG ODN), zymosan, and LPS has also been evaluated at the gene level. First, CpG ODN incubation of gilthead seabream or European sea bass leucocytes resulted in the strong up-regulation of *prx1* transcription. This suggests that gilthead seabream *prx1* transcription is related with innate cytotoxic activity after *in vitro* [60] or *in vivo* [61] stimulation by CpG ODNs, which was increased up to 20- and 4-fold, respectively. Moreover, Japanese flounder specimens injected with stimulatory plasmids rich in CpG motifs and then challenged with *Aeromonas hydrophila* showed that amongst the up-regulated immune-related genes in the spleen, *prx1* was the most up-regulated [62]. Interestingly, other studies have related the modulation of *prx1* expression with LPS administration, the main bacterial PAMP. Thus, the injection of LPS in spotted green pufferfish up-regulated *prx1* (*nkefa*) transcription in the spleen, but down-regulated it in the kidney, liver, heart, brain, gill, gonad, and skin [40], similar to the up-regulation found in the spleen of channel catfish [45]. In contrast, the *in vitro* incubation of gilthead seabream or European sea bass KLs with LPS failed to modulate *prx1* transcription [47]. Interestingly, and in the case of whole bacteria, *in vitro* exposure to *V. anguillarum* up-regulated *prx1* gene expression in gilthead seabream leucocytes, but *Photobacterium damselae* failed to do so, as occurred with both bacteria in the European sea bass leucocytes [47]. Interestingly, Japanese flounder infected with *Edwardsiela tarda* also showed increased *prx1* transcription in the spleen [63]. These data point to the importance of the spleen tissue in the role of *prx1* in fish immunity. It is important to note that spleen is the most important secondary lymphoid tissue in fish [65], where it is highly irrigated and presents very high numbers of erythrocytes, which are among the most productive peroxiredoxin-producing cells in fish [44,47,51] and mammals [13,66]. Moreover, LPS induction of *prx1*/*nkefa* transcription in the spleen is related to anti-bacterial defence and potentially reflects its role as inflammatory cytokines in fish. However, a TLR4 homologue has been found in some fish, while others, such as gilthead seabream or spotted green pufferfish, lack TLR4 [67,68]; thus, the mechanism by which Prxs act in this fish species is uncertain but clearly different from that which occurs in mammalian cells [10,18]. Unfortunately, no study has looked at the interaction between fish Prxs and TLR4, or any other leucocyte surface receptor.

Some authors also relate virus infections with fish Prx1. First, polyinosinic-polycytidylic acid (poly I:C), an analogue to viral RNA and an important viral PAMP, up-regulated *prx1* transcription in European sea bass leucocytes but not in gilthead seabream [47]. In common carp infected with Spring Viremia of Carp Virus (SVCV), *prx1* was up-regulated in the peripheral blood leucocytes (PBLs) and down-regulated in the gills [44]. In rainbow trout, Viral Haemorrhagic Septicaemia Virus (VHSV) infection triggered the up-regulation of *prx1* transcription in blood, HKLs [58], and PBLs [59], as well as in a trout RTS11 cell line [57]. In addition, this *prx1*/*nkefa* up-regulation seems to be concomitant to increased cytotoxic activity [57,59], but further studies are needed to clarify this relation. Moreover, in rainbow trout specimens vaccinated with a proven DNA vaccine for VHSV, whether challenged or not with VHSV, *prx1* expression was increased [58]. When European sea bass and gilthead seabream specimens were infected with a strain of nodavirus (RGNNV), which is highly pathogenic for European sea bass where it mainly affects the brain and retina tissues, but not for gilthead seabream (a reservoir species) [69,70], *prx1* was up-regulated in the head-kidney and brain after 1 day but down-regulated in the brain after 15 and 30 days after infection in the gilthead seabream. However, in the European sea bass, it was significantly up-regulated in the head-kidney at all the infection times but only 15 days after infection in the brain [47]. In both species, the *prx1* up-regulation was concomitant with increased innate cytotoxic activity [71]. In humans, it has been shown that virus-infected patients have increased circulating levels of Prx1 and Prx2 and that their intracellular accumulation blocks virus replication through the down-regulation of the NF-κB pathway [15]. Something similar could be happening in the fish defence against fish viruses and deserves further investigation.

Parasites are important fish pathogens, causing great economic losses in aquaculture, since there are no preventive treatments, such as effective vaccines. Thus, in Atlantic salmon infected with *Neoparamoeba perurans*, the causative agent of the amoebic gill disease (AGD), the transcription of *prx1* was significantly reduced in the gill tissues [48]. In gilthead seabream infected with *Enteromyxum leei*, an important intestinal parasite, *prx1* transcription was increased in the intestine of specimens exposed to the parasite, but parasite-free, but was down-regulated in parasitized specimens [46]. Moreover, as eukaryotic cells, parasites also produce Prxs. It is known that the malaria parasite Prxs also binds to TLR4 and increases the production of immune mediators such as inflammatory interleukins, TNF and nitric oxide (NO) in humans [72,73]. It has been suggested that murine Prx1 and malarial Prx promote polarized Th1 immune responses to produce inflammatory cytokines, while Prx2 and helminth Prx promote polarized Th2 immune responses to produce anti-inflammatory cytokines or induce M2/wound-healing macrophages [10]—a hypothesis that should be evaluated in fish.

Interestingly, all of this information at the gene level is complemented by proteomic studies that further support the role of Prx in immunity. Thus, an antibody was generated against trout Prx1 and used in immunohistochemistry, ELISA, and western blot techniques [56]. Trout head-kidney macrophages incubated for 2 h with LPS or zymosan failed to change the production of Prx1 as evidenced by ELISA analysis, though the incubation of these cells with heat-attenuated *Vibrio ordalii* bacteria resulted in a significant increase in Prx1 production. In addition, the trout RTS11 cell line incubated with LPS increased Prx1 protein expression, but not when it was infected with Infectious Salmon Anemia (ISA) virus, as demonstrated by western blot. In addition, trout infected with the parasite *Caligus rogercresseyi* showed increased Prx1 protein expression when evaluated by immunohistochemistry and western blot in spleen and head-kidney samples [56]. Moreover, in large yellow croaker vaccinated with an inactivated trivalent vaccine (*Vibrio alginolyticus*, *V. parahemolyticus*, and *Aeromonas hydrophila*), a proteomic survey found increased amounts of Prx1 in the spleen [37]. Japanese flounder infected with *Edwardsiela tarda* also showed increased Prx1 in the spleen [63].

Finally, very little is known about the direct impact of fish Prx1 on immunity (Table 2). Recombinant Prx1 in Atlantic salmon demonstrated an antioxidant function [48], but no further characterization was carried out. Recently, Japanese flounder specimens were intramuscularly injected with plasmids coding for Prx1 and then infected with *E. tarda*, resulting in reduced bacterial load in spleen and kidney [63]. The same authors showed that *prx1* knockdown increased the susceptibility of fish to infection, the results as a whole pointing to a clear role of Prx1 on fish immunity. Therefore, more studies using recombinant or isolated fish Prx1 are needed to confirm its direct role in fish immunology and to make a comparison with its counterpart in mammals, the most evolved vertebrates.

### 3.3. Prx2 (NKEF-B) in Fish Immunity

Prx2 plays a similar role in immunity to fish Prx1 (Table 1 and Table 2). First, as occurs with *prx1* transcription, incubation of gilthead seabream and European sea bass head-kidney leucocytes with mitogens failed to regulate *prx2* expression levels. However, LPS injection up-regulated *prx2* in the spleen but down-regulated it in the kidney, liver, intestine, heart, brain, gill, gonad, and skin of spotted green pufferfish [40], as also occurs in the red blood cells of lamprey [38]. CpG ODNs were also able to up-regulate the *prx2* transcription in gilthead seabream and European sea bass leucocytes [47], which was seen to be related to increased innate cytotoxic activity as a result of *in vitro* or *in vivo* CpG stimulation [60,61].

Regarding to the effects of bacteria, infection of miiuy croaker with *V. anguillarum* up-regulated *prx2* gene expression in the kidney, but not in the spleen or liver [36]. In ayu, the *prx2* mRNA levels were up-regulated in the intestine of fish infected with *V. alginolytics* and in all the assayed tissues (gill, brain, heart, kidney, liver, intestine, muscle, and spleen) when infected with *A. hydrophila* [33]. In turbot specimens and in a turbot embryonic cell line (TEC), infection with *V. anguillarum* resulted in increased levels of *nkef* (most probably *prx2*) gene expression [39]. At the protein level, Prx2 was over-produced in the spleen of large yellow croaker vaccinated with an inactivated trivalent vaccine [37] as well as in the liver of ayu specimens infected with *A. hydrophila* [33].

In the case of fish virus, in gilthead seabream specimens infected with NNV, the *prx2* gene was up- or down-regulated, as occurred in the case of the *prx1* gene [47]. However, *prx2* expression in European sea bass was slightly up-regulated in the head-kidney and unaltered in the brain, which was again related to increased cytotoxic activity of HKLs [71]. In common carp infected with SVCV, *prx2* was up-regulated in PBLs, gills, spleen, intestine, and muscle, but down-regulated in kidney and head-kidney [44]. In addition, by using an antibody against carp Prx2, immunocytochemical analysis revealed an increase in cell numbers and staining intensity in the spleen and gill tissues from SVCV-infected fish, although cross-reaction with Prx1 cannot be discarded. In Atlantic salmon, the Prx2 protein was identified in liver by proteome analysis and was found to be down-regulated in specimens exposed to Infectious Hematopoietic Necrosis Virus (IHNV) infection [52].

In parasitic infections, *prx2* transcription increased in gilthead seabream specimens exposed to *Enteromyxum leei* and parasite-free, but unaltered in parasitized specimens [46].

Finally, recombinant lamprey Prx2 protein was active in ROS scavenging and DNA protection *in vitro* [38]. Unfortunately, no other functional study has been performed to establish the immunological role of fish Prx2, although this is expected to be quite similar to the fish Prx1, as occurs in mammals.

### 3.4. Functions of Prx3

Very little information exists about the role of Prx3 in fish (Table 1) or mammalian immunity. The levels of *prx3* gene expression increased in gilthead seabream specimens exposed to the parasite *E. leei* and parasite-free, and was down-regulated in parasitized specimens [46]. In miiuy croaker, *V. anguillarum* infection greatly up-regulated the *prx3* gene expression in the kidney, had less effect in the spleen, and hardly affected the liver [35]. Zebrafish (*Danio rerio*) infected with *A. hydrophila* showed increased *prx3* gene expression and protein production in the gills as indicated by microarray and proteomic assays [55]. Unfortunately, no study has attempted the functional characterization of Prx3 probably because it is more related to ROS metabolism than to immunity, it being the only Prx that is confined to mitochondria.

### 3.5. Functions of Prx4

The role of peroxiredoxin 4 has been studied more in fish (Table 1 and Table 2) than in mammals. *V. anguillarum* infection greatly up-regulated *prx4* gene expression in the liver and kidney of miiuy croaker while in the spleen it was down-regulated [35]; *E. leei* infection, on the other hand, failed to alter the gilthead seabream transcription of *prx4* [46].

A deeper characterization of Prx4 has been performed in large yellow croaker. First, in croaker specimens vaccinated with an inactivated trivalent vaccine, a proteomic survey found increased amounts of Prx4 in the spleen, which was subsequently confirmed by western blot using a specific antibody against croaker Prx4 [37]. Prx4 localized specifically in the rough endoplasmic reticulum and peroxisomes, as occurs in mammals. Functional characterization corroborated its antioxidant activity but also clearly identified its importance in immunity. Thus, when the *prx4* gene expression was knocked out *in vivo* by siRNA, the NF-κB activity increased in the spleen and the expression of genes coding for pro-inflammatory *tnfa* and several chemokines were up-regulated, whilst the anti-inflammatory *il10* gene expression was down-regulated [37]. Interestingly, when fish were injected with the recombinant Prx4 the opposite occurred; that is, NF-κB activity decreased and cytokine gene expression profiles were reverted. Moreover, siRNA increased large yellow croaker mortality upon bacterial infection whilst the recombinant protein protected the fish [37]. Subsequently, it was demonstrated that the removal of the N-terminal antiparallel β-sheet motif eliminates the ability to negatively regulate the NF-κB transcription factor, reduces the antioxidant activity, and abrogates the fish resistance to bacterial infection, indicating the pivotal importance of this motif in its function [64]. In mammals, Prx4 is the only secretable Prx protein. However, contradictory results point to its role in mammalian immunity. Thus, while overexpression in human cells of a Prx4 isoform (AOE_372_) blocked the NF-κB activation mediated by TNF and phorbol ester [74], other studies show that another Prx4 isoform (TRANK) activates NF-κB [75]. Consequently, Prx4 may play a role in the induction, rather than the inhibition, of inflammation. Thus, results obtained in the large yellow croaker are in line with the NF-κB blockade conferring an anti-inflammatory state.

### 3.6. Functions of Prx5

In mammals, recombinant Prx5 binds to TLR4, and is the Prx that induces the initiation of post-ischemic inflammation the most [19]. In fish, few studies have dealt with Prx5 (Table 1). The miiuy croaker infected with *V. anguillarum* showed great up-regulation of the *prx5* gene expression in the liver, kidney and spleen, the highest levels being reached in the kidney [35]. Zebrafish infected with *A. hydrophila* showed increased Prx5 gene and protein levels in gills, as indicated by microarray and proteomic assays [55]. In the case of gilthead seabream exposed to the parasite *E. leei*, *prx5* showed a similar profile to *prx1*, *prx2*, and *prx3* [46].

### 3.7. Functions of Prx6

Prx6 is a bifunctional enzyme with both glutathione peroxidase and acidic Ca^2+^-independent phospholipase A2 activities [76]. However, it is of vital importance in ROS production by phagocyte cells since it interacts and supports the optimal activity of NAPDH oxidase, the main ROS source in phagocytes [23].

At the gene level, *prx6* transcription (Table 1) was unaltered or down-regulated in gilthead seabream specimens exposed to *E. leei*, depending on the absence or presence of the parasite [46]. Rock bream injected with poly I:C or iridovirus showed up-regulated transcription of *prx6* in liver but not in the blood [49]. Similarly, the injection of poly I:C, *V. anguillarum,* or *Streptococcus iniae* in turbot induced a significant up-regulation of the *prx6* gene in the liver and spleen tissues, being highest in the spleen for *V. anguillarum* and in the liver for *S. iniae* [34].

The use of recombinant Prx6 (Table 2) *in vitro* protected against supercolloid DNA damage by metal-catalyzed oxidation in rock bream [49] and protected hepatocytes from cell death induced by hydrogen peroxide incubation in turbot [34], suggesting a potent cytoprotective role against oxidative stress in both species.

## 4. Preliminary Functional Data in European Sea Bass

After confirming that leucocytes showed increased cytotoxic activity [71] and regulate *prx1* and *prx2* transcription in gilthead seabream and European sea bass upon NNV infection [47], our aim turned to expanding our knowledge of their potential immunological role.

### 4.1. Methodology

In brief, gilthead seabream *prx1* (GQ252679) and *prx2* (GQ252680) gene sequences were cloned into the expression vector pcDNA3.1/V5-His-TOPO (Invitrogen). Empty constructions (pcDNA) were also created as controls. The constructions were used to transform into competent *E. coli* DH-5α cells, and plasmids were isolated using the QIAGEN Plasmid Midi kit (Quiagen). Plasmid DNA was sequenced using an ABI PRISM 377 sequencer to verify the sequence and construction.

European sea bass specimens (32 fish of 5–15 g randomly divided into 4 aquaria), maintained in marine aquaria at the University of Murcia, were intramuscularly injected with generated plasmids and NNV [71] as follows: saline + NNV, pcDNA + NNV, *prx1* plasmid + NNV, or *prx2* plasmid + NNV. Plasmids were injected at 1 µg/fish and NNV at 10^6^/fish. After 1 and 7 days, 4 fish per tank were sacrificed, and the brain, the main target tissue for NNV, was sampled. The RNA was isolated, the cDNA created, and the expression of genes were studied by real-time PCR as described elsewhere [47,71]. To evaluate their potential protective role against NNV replication, the expression of the antiviral *mx* (interferon-induced GTP-binding protein Mx) and the viral *cp* (NNV capsid protein) genes was analyzed in the brain as previously published [71,77]. Gene expression was normalized to that of the housekeeping elongation factor 1 alpha (*ef1a*). Animal studies were carried out in accordance with the European Union regulations for animal experimentation and the Bioethical Committee of the University of Murcia.

### 4.2. Results and Discussion

**Figure 2:**
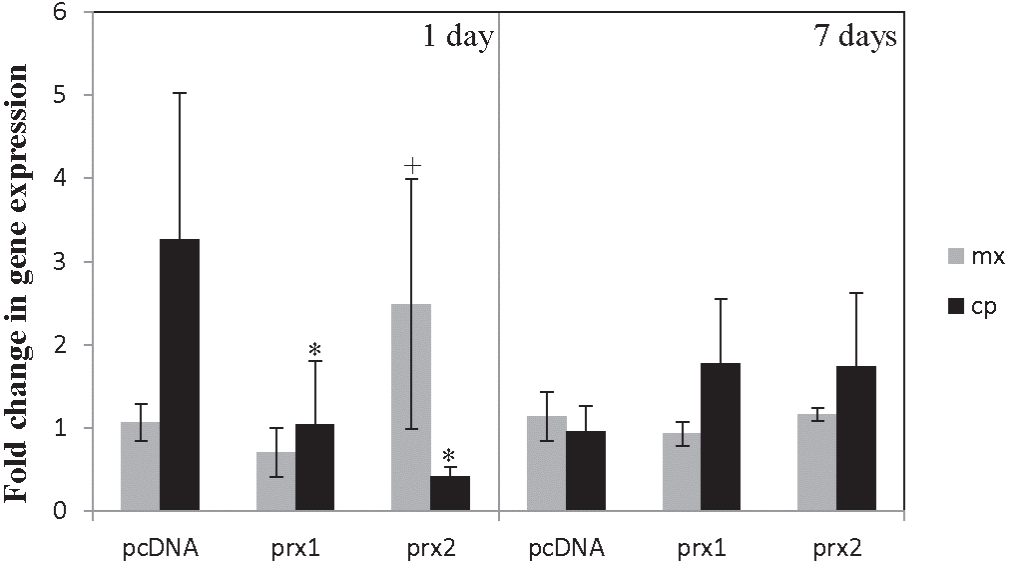
Expression levels of the antiviral *mx* and the nodavirus capsid protein (*cp*) genes in the brain of European sea bass specimens injected with expression plasmids (pcDNA alone or containing the gilthead seabream *prx1* or *prx2* genes) and infected with NNV. Bars represent the mean ± SEM (*n* = 4) referenced to the values found in control fish. Statistical differences (*p* < 0.1, +; *p* < 0.05, *) were analysed by ANOVA.

The data show that *mx* transcription is slightly up-regulated by *prx2* expression in NNV-infected brains (Figure 2). Moreover, after 1 day of infection, the expression of the NNV capsid gene was significantly reduced in the *prx1* and *prx2* injected group but the protective effects were abrogated at 7 days. These data point to the beneficial role of gilthead seabream Prx1 or Prx2 in the resistance of European sea bass to NNV.

Previous results suggest an important role for Prx1 and Prx2 in protection against viral infections. Interestingly, brain from NNV-infected European sea bass showed no alteration in *prx1* or *prx2* gene expression when compared to controls [47], suggesting that they are not involved in immunity against this virus, in sharp contrast to the increased *prx* transcription found in other fish-virus models (see Table 1). Although the precise mechanism is unclear, the intramuscular expression of seabream *prx1* or *prx2* plasmids leads to a transitory resistance in the brain upon NNV infection. Similarly, the intramuscular injection of plasmids encoding genes related to the immune response resulted in decreased viral loads or increased immune response and resistance [78,79]. Our results are also in line with those demonstrating increased bacterial resistance following Prx1 and Prx4 treatment [37,63,64]. Based on this preliminary study, gilthead seabream Prx1 and Prx2 confer short term protection in European sea bass against NNV infection, although further studies are needed to ascertain the role of fish Prxs in immunity.

## 5. Conclusions

Peroxiredoxins are a family of six proteins with an important role in scavenging the ROS produced in cells, although other roles have been attributed to them, especially their involvement in inflammation through binding to TLR4. In fish, Prx1–6 have been identified at both the gene and protein levels with special emphasis on Prx1, Prx2, and Prx4. Their profiles of gene expression and protein production point to a role for Prxs in the immune response against bacterial, viral, and parasitic infections, suggesting a major relevance in immune tissues. Unfortunately, and although fish Prx1, Prx2, Prx4, and Prx6 are known to play a role as antioxidants, little effort has been dedicated to demonstrating their involvement in immunity. In that sense there are few data on the overproduction or knockdown of Prx2 and Prx4 that clearly point to a role for these proteins in fish immunity and suggest that they could play a very similar role to that observed in mammalian species, using similar mechanisms in the process. We have made an effort to relate these proteins to the anti-viral immune response and fish resistance against nodavirus and found that the intramuscular expression of seabream *prx1* or *prx2* plasmids leads to a transitory resistance in the brain of European sea bass upon NNV infection. In our considered opinion, all the data reviewed here confirm that fish Prx have a role in immunity that deserves further study, such as an evaluation of their direct involvement in cytotoxic activity and interaction with TLR4.
